# Photophysics of Distinguished Defect States in Bi_2_O_2_Se Thin Films

**DOI:** 10.1002/nap2.70280

**Published:** 2026-08-28

**Authors:** Xiaoran Niu, Hao Li, Leyang Dang, Ruoying Pang, Xin Meng, Adeela Nairan, Huarui Sun, Jingkai Qin, Usman Khan, Xiaochuan Xu, Guigen Wang, Feng He

**Affiliations:** ^1^ National Key Laboratory of Laser Spatial Information Harbin Institute of Technology (Shenzhen) Shenzhen China; ^2^ Guangdong Provincial Key Laboratory of Aerospace Communication and Networking Technology, Guangdong Provincial Key Laboratory of Integrated Photonic‐Electronic Chip Harbin Institute of Technology (Shenzhen) Shenzhen China; ^3^ Sauvage Laboratory for Smart Materials, School of Materials Science and Engineering Harbin Institute of Technology (Shenzhen) Shenzhen China; ^4^ School of Materials Science and Engineering Institute of Functional Porous Materials, Zhejiang Sci‐Tech University Hangzhou China; ^5^ Shenzhen Key Laboratory for Advanced Materials, School of Materials Science and Engineering Harbin Institute of Technology (Shenzhen) Shenzhen China; ^6^ School of Science and Ministry of Industry and Information Technology, Key Laboratory of Micro‐Nano Opto‐Electronic Information System Harbin Institute of Technology (Shenzhen) Shenzhen Guangdong China

**Keywords:** Bi_2_O_2_Se thin films, defect trapping, pump–probe experiment

## Abstract

As a leading candidate for next‐generation electronics, high‐performance 2D Bi_2_O_2_Se has garnered significant interest in the scientific community. However, the understanding of its defect mechanisms remains elusive, hindering the development of further functionalities. In this study, we utilized Raman spectroscopy and ultrafast pump–probe experiments to investigate Bi_2_O_2_Se thin films prepared via a solution‐based technique. By comparing carrier decay times under low and high fluences in 25 nm‐ and 115 nm‐thick films, we proposed the defect mechanisms associated with two common defects in the thicker film: Se vacancies and Se–Bi antisites, which exhibit shallow donor and deep donor behaviors, respectively. Additionally, Se–Bi antisites became prominent under higher fluences. Our insights into these defect mechanisms offer valuable guidance for defect engineering in high‐power electronics.

## Graphical abstract

An experimental comparison of CVD‐grown Bi_2_O_2_Se thin film and films prepared by OTG solution method with similar electrical properties reveals the defects mechanisms in Bi_2_O_2_Se thin films via OTG solution method, offering defect engineering strategies for high‐power electronics.

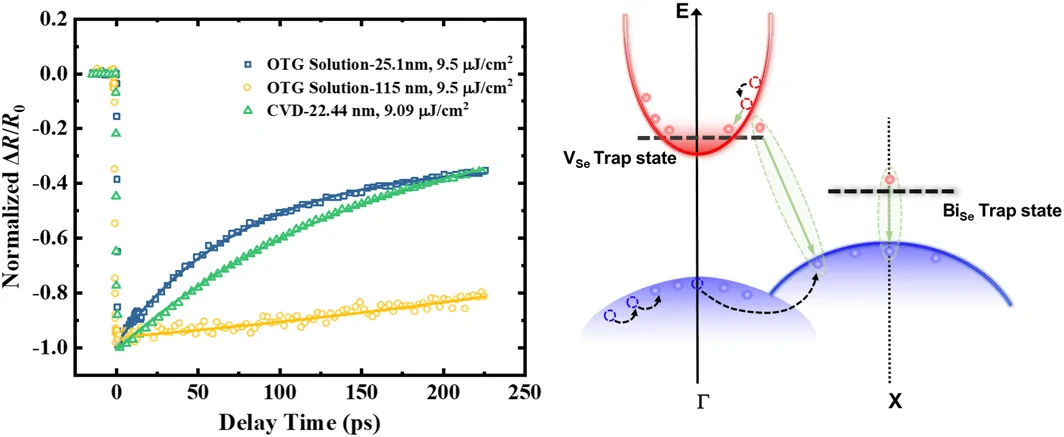

## 1 Introduction

2D Bi_2_O_2_Se, renowned for its air‐stability and exceptional carrier mobility, stands out as a promising candidate for future electronics [1, 2]. Various synthesis methods, including chemical vapor deposition (CVD), physical vapor deposition (PVD), molecular beam epitaxy (MBE), pulsed laser deposition (PLD), and solution‐based techniques, have been explored for Bi_2_O_2_Se thin films [3, 4, 5, 6]. While CVD and PVD methods yield sizable films, up to millimeter‐scale, on mica substrates, their transfer without damage remains challenging due to strong electrostatic forces. Conventional solution transfer techniques, which rely on HF etching, pose a risk of sample degradation [7]. Bi_2_O_2_Se produced by MBE or PLD typically requires costly equipment and ultra‐high vacuum conditions, limiting large‐scale industrial applications [8]. In contrast, solution methods have emerged as practical alternatives for synthesizing Bi_2_O_2_Se and other 2D materials [9, 10, 11, 12]. Previous reports utilized hazardous reactants like Bi(NO_3_)_3_·5H_2_O and selenium powder, with hydrazine hydrate (N_2_H_4_) as a reducing agent [13]. Recently, Dang et al. introduced an organic ion template‐guided solution growth method (OTG), offering a safer approach to producing high‐quality Bi_2_O_2_Se thin films with adjustable properties like area, thickness, and oxygen defects [3].

As suggested by Righetto et al., the solution‐processing method results in a significant number of defects (approximately 10^16^–10^17^ cm^−3^), predominantly characterized by shallow traps with a smaller population of deep trap sites [14]. Nevertheless, the electrical properties, such as mobility, exhibit comparable performance between films produced via CVD and OTG methods [3, 15]. However, the precise influence of these defects on carrier transport properties remains elusive. Various defect mechanisms contribute to this ambiguity, including defect‐assisted recombination, which reduces carrier lifetime, and defect‐trapping and de‐trapping mechanisms, which prolongs carrier lifetime [14, 16, 17]. Chen et al. found a 50‐fold increase in carrier recombination rates in MBE‐grown MoTe_2_ and MoSe_2_ films compared to exfoliated ones, employing ultrafast laser pump–probe spectroscopy [18]. Yuan et al. noted higher exciton mobility in exfoliated samples versus CVD‐grown counterparts [19]. Katsiaounis et al. observed variations in substrate‐induced phonon dynamics, influencing the phonon lifetime of monolayer graphene in exfoliated samples compared to thicker crystals, while CVD‐grown graphene displayed shorter lifetimes due to structural defects [20]. Debates persist regarding the scattering mechanisms arising from defects in Bi_2_O_2_Se. Fu et al. utilized density functional theory (DFT) to demonstrate that the donor levels of two prominent defects in Bi_2_O_2_Se, namely Se vacancies (*V*_Se_) and Se–Bi antisites (Bi_Se_, one Se atom substituted by Bi), reside above the bottom of the conduction band [21]. Li et al. noted, through DFT calculations, that both O vacancies (*V*_O_) and *V*_Se_ act as shallow donors positioned close to the conduction band minimum (CBM), whereas Bi_Se_ serves as a deep donor center [22]. Meanwhile, Wei et al. contended that *V*_Se_ level lies above the CBM, enhancing carrier mobility by generating free carriers, while Bi_Se_ level resides in the middle of the bandgap, thereby reducing carrier lifetime by functioning as electron‐hole traps and recombination centers [23]. Although previous DFT studies have proposed several intrinsic defects, including *V*_Se_, *V*_O_, and Bi_Se_, their transition levels and their influence on carrier dynamics remain under debate. Consequently, direct experimental evidence linking specific defects to ultrafast carrier relaxation is still lacking.

In this study, we employed pump–probe measurements to investigate the impact of defects on carrier decay time in Bi_2_O_2_Se thin films produced via the CVD and OTG methods. Our measurements reveal distinct carrier relaxation behaviors that are consistent with shallow‐ and deep‐level defect‐assisted recombination. By combining pump–probe spectroscopy, Raman measurements, and previous DFT calculations, we propose that these behaviors can be tentatively attributed to *V*_Se_‐ and Bi_Se_‐related defects.

## 2 Results and Discussion

### 2.1 The Role of Defects in OTG‐Prepared Bi_2_O_2_Se Film

The surface morphologies (in Figure 1a,b) of Bi_2_O_2_Se films indicate that the thickness of the CVD‐grown sample (denoted as Sample C1) is 22.44 nm, with a surface roughness smaller than 5 Å, while the thickness of the sample synthesized via the OTG solution method (denoted as Sample O1) is 25.1 nm, with a surface roughness smaller than 10 Å. To explore the impact of various growth methods on carrier dynamics, we conducted degenerate 800 nm pump–800 nm probe measurements with Oscillator (80 MHz, 35 fs) on these two samples. Specific experimental parameters can be found in our previous research [24]. Figure 1c illustrates the normalized differential reflectivity signals of these two samples under the low pump fluence (∼9 μJ/cm^2^). Both signals exhibit a rapid decrease at 0 ps, followed by a slow recovery. To analyze the relaxation process, we applied a single exponential decay fitting function, as shown in Equation (1) (*n* = 1) to extract corresponding values:

(1) $$\frac{\Delta R}{R_{0}}=\sum A_{n}e^{-\frac{t}{\tau_{n}}}+y_{0},$$

where Δ*R* represents the transient reflectance change at time *t*, *R*_0_ is reflectance without pump, *A*_*n*_ is the amplitude of the corresponding process, *τ*_*n*_ is the decay time of different decay channels, and *y*_0_ is an offset term.

**FIGURE 1:**
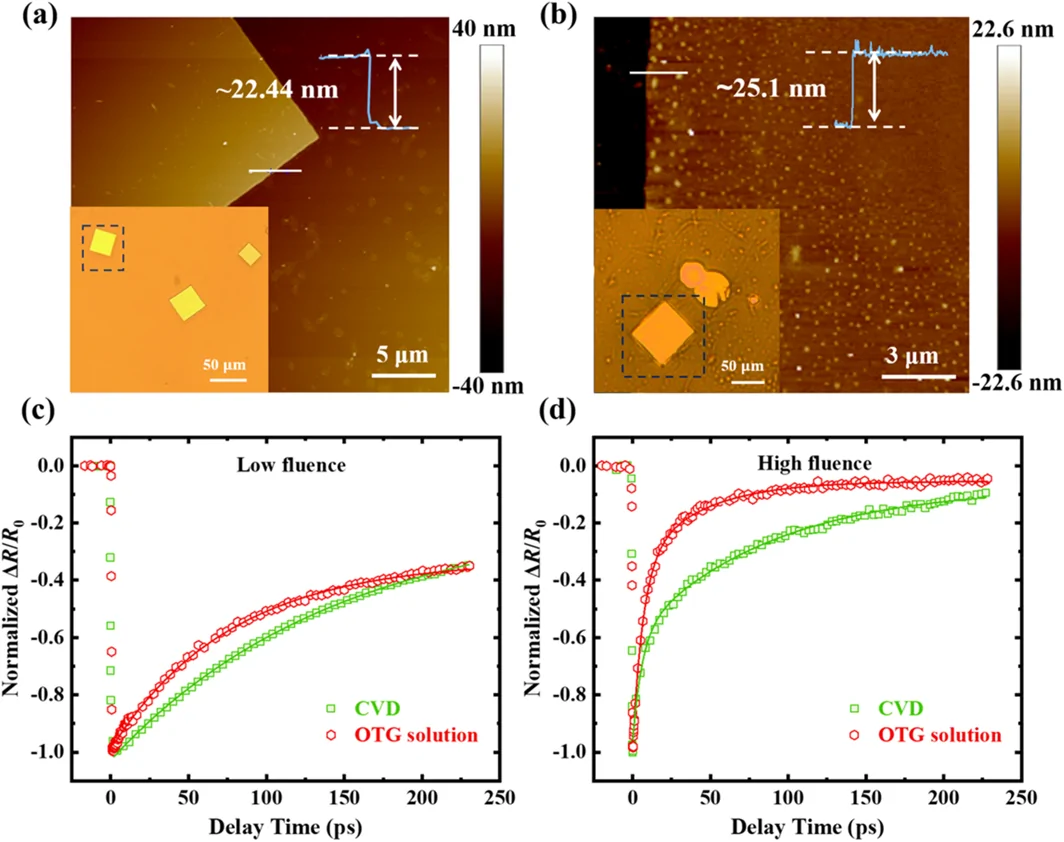
Atomic force microscope (AFM) images of Bi_2_O_2_Se film synthesized by (a) CVD and (b) OTG solution methods, with thicknesses of 22.44 nm on mica and 25.1 nm on SiO_2_ substrates, respectively. The insets in the lower left corner display corresponding optical microscopy images. (c) Normalized transient differential reflectivity signals Δ*R/R*_0_ of the sample in (a) at a pump fluence of 9.09 μJ/cm^2^, as well as the sample in (b) at a pump fluence of 9.5 μJ/cm^2^. The solid lines represent single exponential fitting. (d) The corresponding normalized transient differential reflectivity signals Δ*R/R*_0_ at a pump fluence of 2.2 mJ/cm^2^. The solid lines represent double exponential fitting. Green and red colors denote Bi_2_O_2_Se thin films grown by CVD and OTG solution methods, respectively.

The extracted decay time of Sample C1 is ∼140.35 ± 0.77 ps, which is 1.8 times the extracted value of Sample O1 (∼79.65 ± 0.89 ps), and we attribute this decay channel to the recombination of electron and hole pairs (e‐h recombination) [24]. To check the high power performance, we conducted degenerate 800 nm pump–800 nm probe experiments with Amplifier (5 kHz, 35 fs) at high pump fluence, 2.2 mJ/cm^2^, as shown in Figure 1d. In our previous paper, we demonstrated that the Δ*R/R*_0_ signal comprises two decay components under high fluences. The faster component *τ*_1_, lasting merely a few picoseconds, is attributed to Auger recombination, while the slower component *τ*_2_, lasting several tens of picoseconds, corresponds to electron‐hole (e‐h) recombination [24]. The extracted *τ*_2_ of the Sample C1 (∼89.74 ± 2.16 ps) is 2.3 times higher than that of the Sample O1 (∼38.47 ± 1.25 ps).

It is observed that under both low and high fluence measurement conditions, the e‐h recombination decay time of the sample synthesized via the OTG solution method is shorter than that of the sample grown by CVD. This suggests the presence of mechanisms causing decay time shortening in the OTG solution method, possibly related to defects. At this stage, however, the microscopic origin of these defects cannot be determined solely from pump–probe measurements. Other factors, including substrate effects, morphology, or interface scattering, may also influence the carrier dynamics. Therefore, additional structural characterization is required. In the hydrothermal method, accurately and quantitatively controlling the reaction conditions is challenging. Consequently, impurities and incomplete reactions may occur during the reaction process, ultimately resulting in defects within the synthesized sample. Such impurities and defects in semiconductors can serve as e‐h recombination centers, accelerating the relaxation of photoexcited carriers, and shortening lifetime [25]. Despite comparable electrical properties observed between films produced via CVD and OTG methods, as highlighted in previous studies [3, 15], Righetto et al. proposed that defect tolerance may not apply to hot carriers in perovskite materials [14]. Hence, we provisionally attribute the observed decay time difference to the inherent defects present in the samples synthesized by OTG solution method. Surprisingly, as illustrated in the Supporting Information S1: Figure S1, at a similar pump fluence (∼9 μJ/cm^2^), the 115 nm‐thick film synthesized by OTG solution method exhibits a longer decay time of 227 ps compared to Sample C1, contrary to our expectations. To understand this discrepancy, we further compared the samples using Raman spectroscopy and pump–probe technique. This comparison aims to uncover the factors influencing the observed decay time difference and identify the underlying mechanism.

### 2.2 The Raman Characterization of Bi_2_O_2_Se Film

As shown in Table 1, for simplicity, we will use Sample C1, O1, and O2 to stand for Bi_2_O_2_Se films with different thicknesses and different synthesized methods in the following.

**TABLE 1:** A simple representation of Bi_2_O_2_Se films with different thicknesses and synthesis methods.

Sample	Thickness (nm)	Substrate	Synthetic method
C1	22.44	Mica	CVD
O1	25.1	SiO_2_	OTG solution
O2	115	SiO_2_	OTG solution

Figure 2a indicates that the thickness of the sample synthesized via the OTG solution method is 115 nm, with a surface roughness smaller than 10 Å (Supporting Information S1: Figure S2). From the Raman spectra (in Figure 2b), we observed similar A_1g_ modes for Bi_2_O_2_Se films across five different films synthesized by OTG solution method, with film thicknesses varying from 25.1 to 115 nm. Through Lorentz fitting, we obtained the A_1g_ phonon frequency and width as a function of film thickness, as depicted in Figure 2c,d, respectively. Additionally, for comparison, the frequency and width of A_1g_ mode of the samples grown by CVD [24] are also included in Figure 2c,d.

**FIGURE 2:**
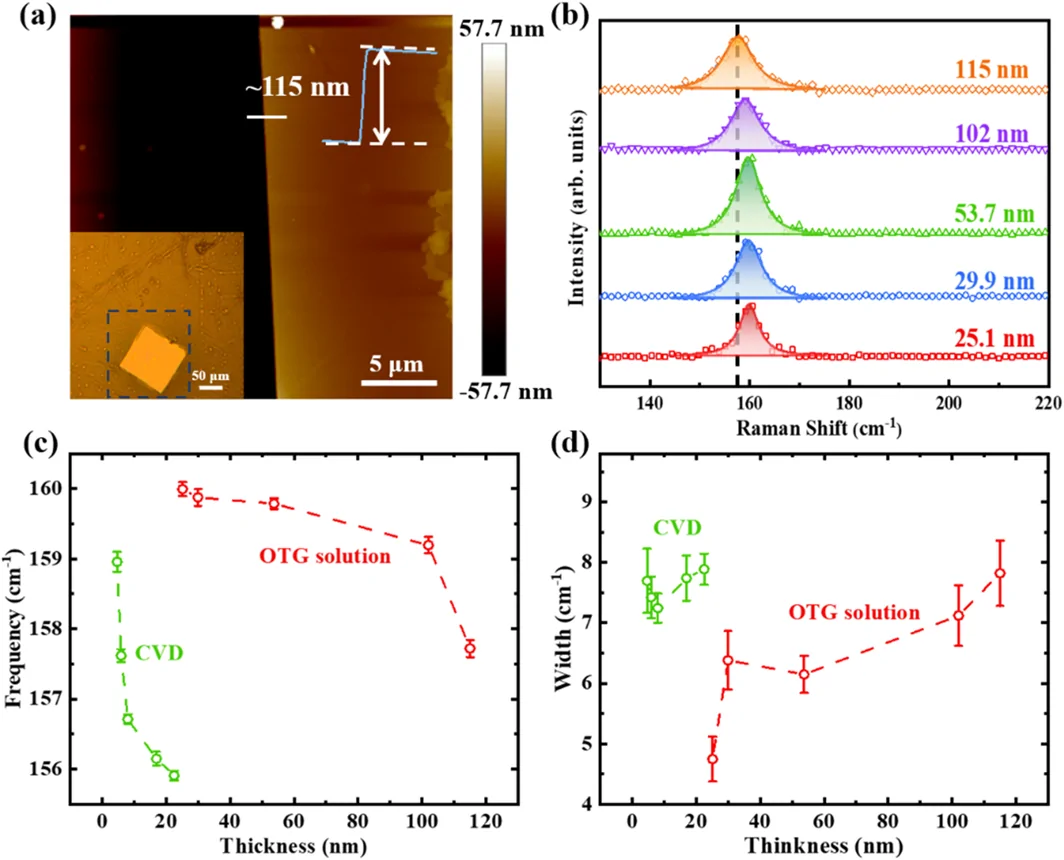
(a) AFM image of 115 nm‐thick Bi_2_O_2_Se film on SiO_2_ synthesized by OTG solution method. (b) Thickness‐dependent Raman spectra (empty dots) of Bi_2_O_2_Se thin films synthesized via OTG solution method and the corresponding Lorentz fitting (solid lines). The dashed lines are included to guide the eye, representing the A_1g_ mode in Bi_2_O_2_Se. The fitted frequency (c) and width (d) of A_1g_ modes are plotted as a function of film thickness. Specifically, the green circles represent Bi_2_O_2_Se grown on mica substrate by CVD method, whereas the red circles correspond to Bi_2_O_2_Se synthesized on SiO_2_ substrate via the OTG solution method.

For samples synthesized by OTG solution method, the A_1g_ phonon frequency exhibits a blue‐shift in thinner films, while the width increases as the thickness increases, as shown in Figure 2c,d. The frequency trend is consistent with previous reports [5, 26], suggesting that it may be induced by an out‐of‐plane compressive strain at the Bi_2_O_2_Se/SiO_2_ interface [27]. As depicted in Figure 2d, the observed increase in Raman linewidth suggests reduced crystallinity or increased structural disorder. Although Raman spectroscopy cannot identify specific defect species, these observations are consistent with an increased defect density in thicker OTG‐grown films [28].

As illustrated in Figure 2c, the A_1g_ phonon frequency of Sample O1 is higher than that of Sample C1. This difference can be attributed to stain induced by the interface, aligning with the findings of Hossain et al., who reported that the compressive strain at the Bi_2_O_2_Se/SiO_2_ interface is greater than that at the Bi_2_O_2_Se/mica interface [29]. In addition, as depicted in Figure 2d, for samples grown by CVD, the lack of dependence of A_1g_ phonon width on film thickness suggests consistent film quality across different thicknesses and no evidence for increased defect‐related broadening, suggesting consistent crystalline quality across the investigated CVD‐grown films. No evidence of thickness‐dependent defect‐related carrier trapping is observed within this thickness range. This indicate that quantum‐confinement effects become negligible above 24.44 nm [24]. Moreover, the narrower peak width of the samples synthesized via OTG Solution method further indicates that the substrate does not play a dominant role here. Combined with our low‐fluence pump–probe results showing that the decay time of the Sample O2 is significantly longer than that of the Sample O1, we can exclude boundary scattering. Additionally, AFM measurements (Supporting Information S1: Figure S2) show surface fluctuations below 10 Å for all OTG samples, indicating comparable surface morphology. Furthermore, pump–probe measurements performed at three different positions on each sample (Supporting Information S1: Figure S3) show excellent reproducibility, suggesting that local morphological variations have a negligible influence on the measured carrier dynamics. Considering that the optical penetration depth greatly exceeds the measured surface roughness, morphology‐related surface effects are unlikely to dominate the observed decay behavior. Consequently, surface roughness is unlikely to account for the large lifetime difference between O1 and O2. Therefore, we can preliminarily propose that the difference in the decay time of samples synthesized by the OTG solution method is mainly caused by defects.

### 2.3 Defect Mechanisms Under High Fluences

The influence of defects on charge carrier dynamics and the underlying mechanisms governing electron‐hole recombination are indeed intricate and not fully understood. While we initially suspected that samples prepared by the OTG solution method might exhibit accelerated decay due to inherent defect states, results from Supporting Information S1: Figure S1 contradict this assumption, with Sample O2 showing a longer decay time than Sample O1. To better understand the defect mechanism in Bi_2_O_2_Se thin films, we conducted high‐fluence pump–probe experiments on Sample O1 and Sample O2.

Figure 3a,b display the Δ*R/R*_0_ of Sample O1 and Sample O2 at high pump fluences ranging from 2.2 to 7.5 mJ/cm^2^, respectively. Here, we define the relative amplitude $a_{i}=A_{i}∕\sum_{1}^{3}A_{i}$. The decay time and corresponding relative amplitudes as a function of pump fluences are plotted in Figure 3c,d, respectively. The uncertainties reported in this work correspond to fitting errors of the transient reflectivity change signals and do not include sample‐to‐sample variations associated with the synthesis process. Although Raman spectroscopy and pump–probe measurements performed at multiple positions demonstrate good spatial uniformity and measurement reproducibility, systematic statistical measurements on independently synthesized films would provide a more comprehensive assessment of fabrication‐induced variability. Such investigations will be the subject of future work. Three main findings emerge. First, as shown in Figure 3c, the extracted decay time decreases as the fluence increases in both samples, consistent with the trend observed in the study by Li et al. [16]. It is noteworthy that the decay times, *τ*_1_ and *τ*_2_, are significantly higher in Sample O2 compared to Sample O1. The longer decay time is not attributed to a ferroelectric transition, as demonstrated in our previous research, which indicates that this transition should occur in thinner (< 8 nm) Bi_2_O_2_Se films [24]. We hypothesize, tentatively, that a specific defect within the Sample O2 significantly contributes to extending its overall decay time.

**FIGURE 3:**
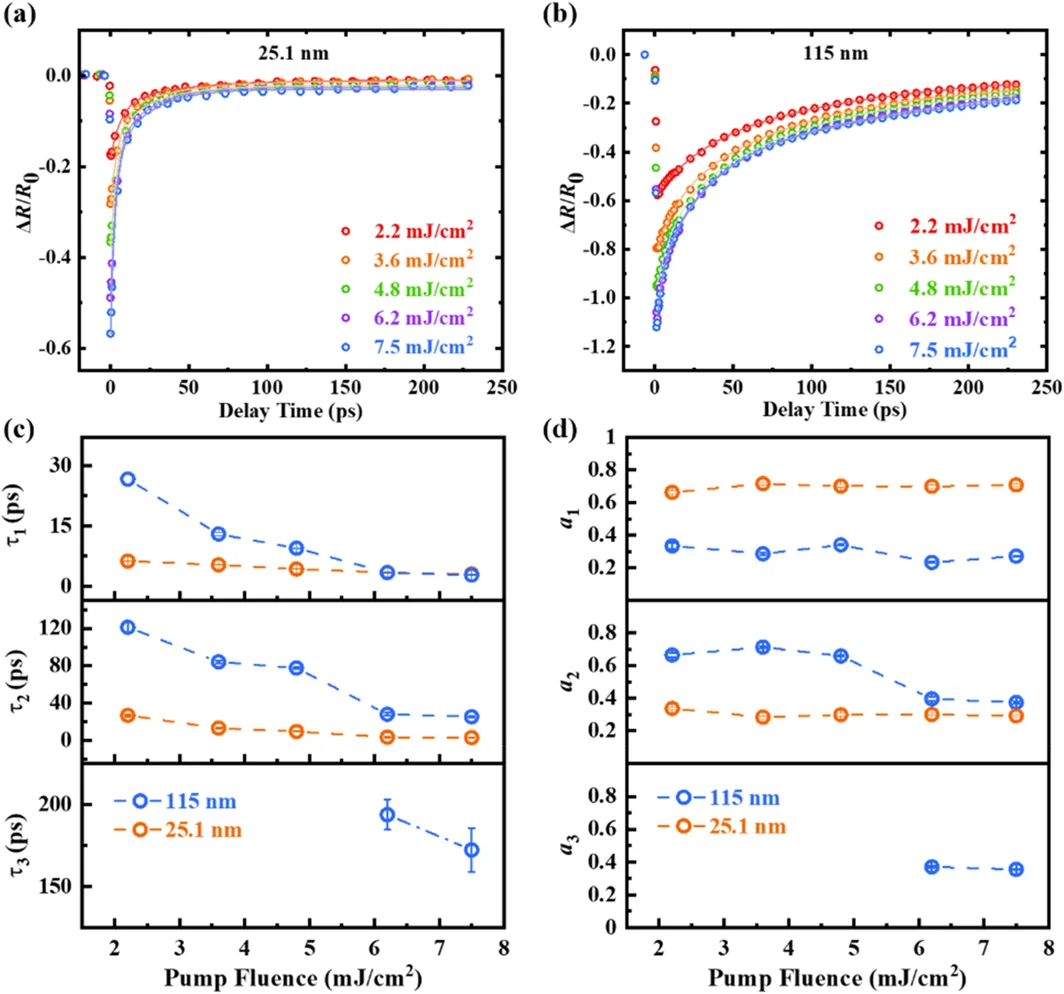
Transient reflectivity change for (a) Sample O1 and (b) Sample O2 at pump fluences ranging from 2.2 to 7.5 mJ/cm^2^, along with their corresponding exponential fittings (solid lines). (c) The extracted decay time as a function of pump fluence. (d) The corresponding relative amplitudes as a function of pump fluences. The orange and blue circles represent Sample O1 and Sample O2, respectively.

Second, as depicted in Figure 3d, it becomes evident that the fast component *a*_1_ predominates in Sample O1. However, as the fluence increases, the relative proportions of the fast *a*_1_ and slow *a*_2_ components remain essentially unchanged. This represents that the two relaxation channels in Sample O1 can maintain a relatively balanced state as the photoexcited carrier density increases. In contrast, in Sample O2, the proportion of the slow component *a*_2_ is significant. As the photoexcited carrier density rises, the proportion of *a*_2_ remains relatively stable until the fluence surpasses 5 mJ/cm^2^. Beyond this threshold, the slow component *a*_2_ transitions from stability to a sharp decline.

Third, as fluence increases above 5 mJ/cm^2^, a third decay time component, *τ*_3_, emerges in Sample O2. Consequently, both Sample O1 and Sample O2 film exhibit similar decay constants, *τ*_1_ and *τ*_2_. This observation suggests activation of an additional relaxation channel in Sample O2 that becomes dominant only under extremely high pump fluence with a recombination process of approximately 200 ps, 4 times longer than *τ*_2_.

Defects have been demonstrated to be effective traps for capturing photoexcited carriers. As suggested by Righetto et al., structural defects are commonly presented in solution‐processed perovskite films, consisting of numerous shallow trap sites and fewer deep trap sites [14, 30]. In Bi_2_O_2_Se, intrinsic point defects include vacancies (*V*_Bi_, *V*_O_, and *V*_Se_), interstitials (*I*_Bi_, *I*_O_, and *I*_Se_), and substitutions (Bi_O_, Bi_Se_, O_Bi_, Se_Bi_, O_Se_, and Se_O_) [21, 22, 23]. X‐ray photoelectron spectroscopy (XPS) confirmed that the atomic ratio of Bi to Se in the samples grown by CVD was 2:1, indicating sufficient stoichiometry for nanoribbon formation [31]. However, in the sample synthesized by OTG solution method, the atomic ratio of Bi to Se is 2.02:0.98, indicating a deficiency of Se (Supporting Information S1: Figure S3) [3]. The observed Se deficiency indicates that selenium‐related defects are likely present. DFT calculations show that under Se‐poor conditions, Bi_Se_ and *V*_Se_ defects can form easily as donors due to a low formation energy, making them plausible candidates for the observed carrier dynamics. Wei et al. calculated that the transition energy level of *V*_Se_ defects lies 0.55 eV above the conduction band minimum (CBM) [23]. Despite doubts about *V*_Se_ defects being above the CBM, they can still be regarded as shallow defects with a transition energy near the CBM. These shallow *V*_Se_ defects can serve as nonradiative e‐h recombination centers, facilitating the relaxation of photoexcited carriers and resulting in a shorter decay time. Given these premises, we propose that there are *V*_Se_ defects in the Sample O1, resulting in a shorter decay time *τ*_2_ than Sample C1.

Additionally, previous DFT calculations have proposed that the transition energy level of Bi_Se_ is located 0.62 eV above the valence band maximum (VBM), in the middle of the Bi_2_O_2_Se band gap, exhibiting a deep level characteristic. Studies have revealed that carriers captured by deep defects exhibit a longer lifetime compared to those captured by shallow defects [32, 33]. The research conducted by Dou et al. indicates that the imbalance between the rates of capture and release of photoexcited carriers by point defect states results in an extended carrier lifetime in 2D ReX_2_ (X = S, Se) [34]. Similarly, the study by Musicenko et al. demonstrated that the trapping time for deep defects (greater than 10^4^ μs) in perovskite is much longer than that for shallow defects (approximately 100 μs) [35], indicating that the recombination process involving deep defects requires more time. Considering the Se‐deficient composition together with previous DFT studies, this channel with decay time *τ*_3_ in Sample O2 is tentatively assigned to the Bi_Se_ defect‐related trapping. Nevertheless, the present measurements cannot exclude contributions from oxygen‐related defects. Due to the alternating composition of [Bi_2_O_2_] and [Se] layers in Bi_2_O_2_Se, and the spatial separation of the [Se] layer and the [Bi_2_O_2_] layer responsible for electron transport, significant overlap of wave functions between the [Bi_2_O_2_] and [Se] layers occurs only under high‐intensity excitations, leading to the activation of Bi_Se_ defects.

At fluences below 5 mJ/cm^2^, the combined influence of two defects significantly impacts the relaxation of photoexcited carriers. However, as the fluence increases, the effect of Bi_Se_ gradually becomes apparent, with *τ*_3_ only appearing at high fluence, further confirming this fact. Additionally, when the fluence exceeds 5 mJ/cm^2^, the comparable amplitudes of *a*_2_ and *a*_3_ suggest a similar quantity of Bi_Se_ and *V*_Se_ defects participating in the relaxation of photoexcited carriers. However, Bi_Se_ has a deeper defect level compared to *V*_Se_, resulting in a larger decay constant *τ*_3_ than *τ*_2_. Meanwhile, at the fluences less than 5 mJ/cm^2^, the competition between these two defects leads to a larger *τ*_2_ of Sample O2 compared to Sample O1.

Several mechanisms could contribute to the observed carrier dynamics, including substrate coupling, dielectric screening, carrier diffusion, morphology, grain boundaries, trap distributions, and defect‐assisted recombination. Substrate effects are unlikely because O1 and O2 were fabricated on the same substrate. AFM measurements reveal surface roughness below 10 Å for both OTG samples (Supporting Information S1: Figure S2), while narrower Raman peak width of the samples synthesized via OTG solution method further indicates that the substrate does not play a dominant role here. In addition, dielectric screening may influence the absolute carrier lifetime but is unlikely to explain the emergence of an additional decay channel (*τ*_3_) observed only in Sample O2 under high pump fluence. Therefore, although these mechanisms cannot be completely excluded, the observations are most consistent with defect‐assisted relaxation involving shallow and deep traps.

Therefore, we propose the dominant carrier relaxation channels as depicted in Figure 4. Figure 4a,b illustrate the dynamics of photoexcited carriers in the Bi_2_O_2_Se film synthesized by CVD and OTG solution methods, respectively. After the optical excitation at 800 nm, electrons and holes are created in the conduction band and valence band, respectively. In the comparable lower‐defect‐density film grown by CVD, as shown in Figure 4a, photoexcited carriers undergo relaxation through Auger recombination within a few picoseconds and e‐h recombination processes within 100 ps. In contrast, for the 25.1 nm‐thick Bi_2_O_2_Se film synthesized by the OTG solution method, depicted in Figure 4b, following the initial intraband relaxation, the photoexcited carriers naturally accumulate at the CBM or the VBM. Subsequently, partial photoexcited carriers relax through Auger recombination within 10 ps, while the majority of these photoexcited carriers relax via shallow defect‐assisted e‐h recombination within 40 ps, tentatively assigned to *V*_Se_. However, in the 115 nm‐thick Bi_2_O_2_Se film, a proposed Bi_Se_‐related deep trapping channel contributes to a significantly longer carrier decay time compared to the 25.1 nm‐thick film. During the relaxation process, most of the photoexcited carriers are captured by *V*_Se_ and Bi_Se_ defects, and the subsequent 100 ps relaxation process is attributed to these two defect‐assisted e‐h recombinations. As the excitation intensity increases further, the Bi_Se_ defect gradually becomes more prominent. Located at a deeper energy level compared to the *V*_Se_ defect, carriers trapped by the Bi_Se_ defect take a longer time to be detrapped, resulting in a decay time *τ*_3_ associated with Bi_Se_ defect‐assisted e‐h recombination that is 4 times longer than *τ*_2_. The schematic illustrates the dominant relaxation pathways inferred from the experimental observations and should not be interpreted as implying the complete absence of defects in the CVD‐grown film.

**FIGURE 4:**
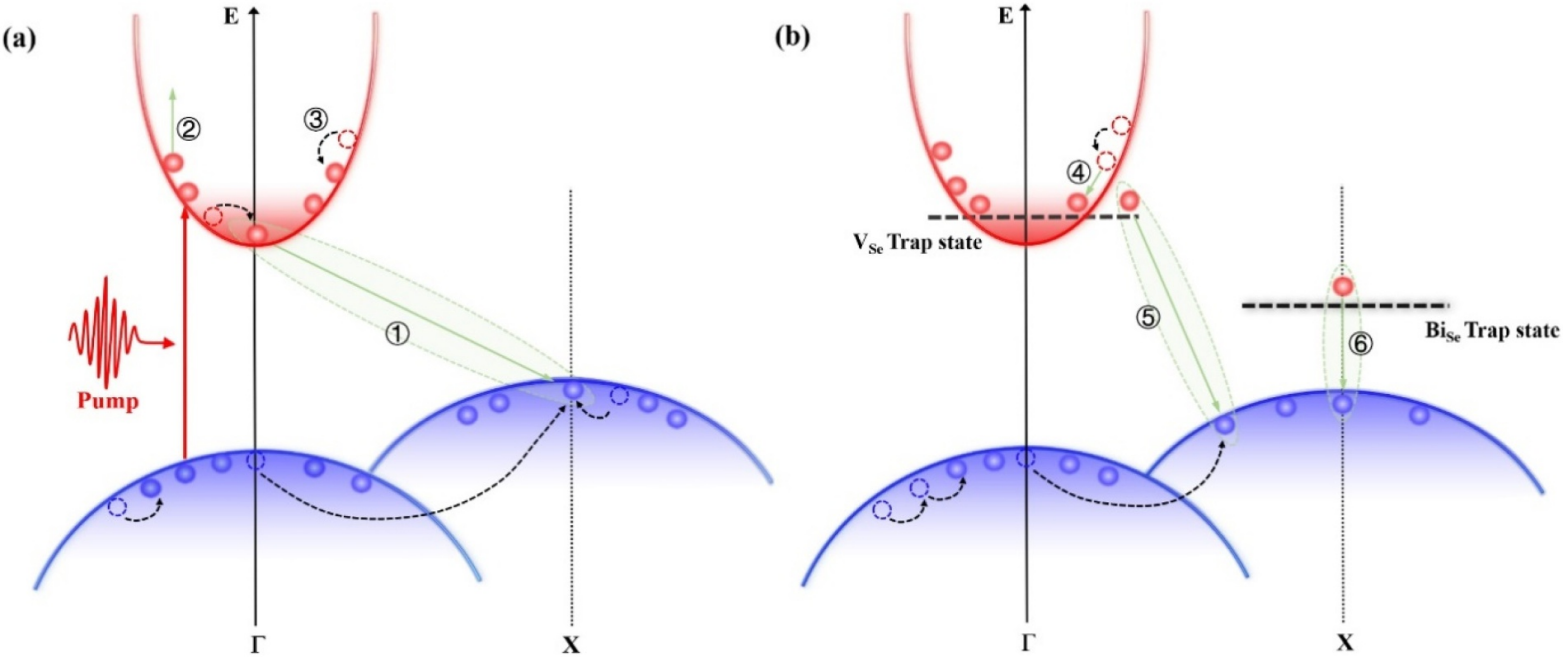
Schematic diagrams illustrating the proposed dominant carrier relaxation channels in Bi_2_O_2_Se film grown by CVD (a) and Bi_2_O_2_Se film synthesized via OTG solution method (b), respectively. The red upward arrow indicates that the electrons in the valence band are excited to the conduction band by absorbing pumping photons, leaving holes in the valence band. The red and blue spheres represent electrons and holes, respectively, while the gray and black dashed lines represent shallow defects caused by *V*_Se_ and deep defects induced by Bi_Se_, respectively. ① e‐h non‐radiative recombination; ② Auger recombination; ③ electron‐phonon scattering; ④ defect‐induced trapping of photoexcited carriers; ⑤ *V*_Se_ defect‐assisted e‐h recombination; ⑥ Bi_Se_ defect‐assisted e‐h recombination.

Although the combined pump–probe, Raman, AFM, XPS composition analysis, and previous DFT calculations consistently support the proposed defect model, direct identification of the microscopic defects requires complementary techniques such as STEM‐EELS, positron annihilation spectroscopy, electron paramagnetic resonance, or spatially resolved XPS. Therefore, the present work proposes a physically consistent defect‐assisted carrier relaxation model rather than definitive microscopic identification.

## 3 Conclusions

In summary, our study delves into the ultrafast carrier dynamics of Bi_2_O_2_Se thin films synthesized via the OTG method. We observed two distinct types of defects in the thicker film, each exerting contrasting effects on carrier decay time. Remarkably, at high fluence, these defects contribute comparably to the carrier decay time. By integrating DFT calculation results from the literature, we proposed these two dominant defect‐assisted relaxation channels as *V*_Se_ defects, associated with shorter decay time, and Bi_Se_ defects, associated with longer decay time. Our findings offer new insights into the influence of defects in Bi_2_O_2_Se synthesized via the OTG solution method, bearing significant implications for future application of Bi_2_O_2_Se.

## 4 Experimental Section

OTG solution synthesis of Bi_2_O_2_Se nanoplates: The synthesis of high‐quality and large‐sized ultrathin 2D Bi_2_O_2_Se was effectuated through an organic ion template‐guided solution growth method, as previously reported by us [3]. In brief, this process involves dissolving ammonium bismuth citrate (Bi(NH_3_)_2_C_6_H_7_O_7_) and sodium selenite (Na_2_SeO_3_) in deionized water. Following a one‐step hydrothermal reaction, the ultrathin 2D Bi_2_O_2_Se nanosheets can be obtained after washing through water or alcohol.

Optical characterizations: The thickness of Bi_2_O_2_Se nanosheets was measured by atomic force microscopy (AFM Bruker Dimension Icon, Germany). Raman results were collected on a Renishaw InVia Raman Microscope system with a 532 nm excitation laser in backscattering geometry.

Degenerate pump–probe system: The laser system is based on a Spectra Physics laser. The laser pulse is split into pump and probe pulses using a beam splitter. These pulses are then focused onto the sample through an objective lens. The pump beam is modulated by a mechanical chopper, while a lock‐in amplifier (Stanford Research Systems, SR860) measures the voltage output of a balanced photodiode (Thorlabs, PDB210A/M) receiving the reflected probe beam. The differential reflectivity (Δ*R/R*_0_ = (*R*−*R*_0_)/*R*_0_) is recorded as a function of pump–probe delay to reveal carrier dynamics.

At low pump fluences, the light source originates from an oscillator (Maitai SP, 800 nm, 35 fs, 84 MHz). The pump and probe beam spot diameters measure approximately 50 and 18 μm (1/e^2^), respectively, while the pump pulses are modulated at a frequency of 2 kHz.

However, under high fluence measurement conditions, the light source was switched to an amplifier (Ascend, 800 nm, 35 fs, 5 kHz). In this scenario, the pump and probe beam spot diameters are approximately 13 and 10 μm (1/e^2^), respectively. Additionally, the pump pulses are modulated at a frequency of 585 Hz.

## Author Contributions

**Xiaoran Niu:** conceptualization, data curation, writing – original draft, investigation, validation. **Hao Li:** conceptualization, data curation, writing – review and editing, software. **Leyang Dang:** conceptualization. **Ruoying Pang:** data curation, writing – review and editing. **Xin Meng:** data curation, validation. **Adeela Nairan:** conceptualization, writing – review and editing. **Huarui Sun:** data curation, methodology. **Jingkai Qin:** conceptualization. **Usman Khan:** conceptualization, writing – review and editing. **Xiaochuan Xu:** conceptualization, writing – review and editing. **Guigen Wang:** conceptualization, formal analysis, funding acquisition, supervision, writing – review and editing. **Feng He:** conceptualization, data curation, formal analysis, funding acquisition, project administration, resources, supervision, writing – review and editing, writing – original draft.

## Conflicts of Interest

The authors declare no conflicts of interest.

## Supporting information


Supporting Information S1

## Data Availability

The data that support the findings of this study are available on request from the corresponding author. The data are not publicly available due to privacy or ethical restrictions.
