# Supplementary material for: Photophysics of Distinguished Defect States in Bi2O2Se Thin Films
Source: Nanophotonics. 2026 Aug 28;15(17):e70280. doi: 10.1002/nap2.70280 (PMC13672171; doi:10.1002/nap2.70280)
Supplement: Supplementary file 1 [file NAP2-15-e70280-s001.docx]

Supporting Information

Photophysics of Distinguished Defect States in Bi_2_O_2_Se Thin Films

*Xiaoran Niu, Hao Li, Leyang Dang, Ruoying Pang, Xin Meng, Adeela Nairan, Huarui Sun,* *Jingkai Qin, Guigen Wang, Usman Khan,* *Xiaochuan Xu, Feng He^*^*

X. Niu, H. Li, R.Pang, X. Meng, F. He

National Key Laboratory of Laser Spatial Information, Harbin Institute of Technology (Shenzhen), Shenzhen 518055, China

Guangdong Provincial Key Laboratory of Aerospace Communication and Networking Technology, Guangdong Provincial Key Laboratory of Integrated Photonic-Electronic Chip, Harbin Institute of Technology (Shenzhen), Shenzhen, China 518055
E-mail: hefeng2020@hit.edu.cn

A. Nairan, U. Khan

Institute of Functional Porous Materials, School of Materials Science and Engineering, Zhejiang Sci-Tech University, Hangzhou 310018, P. R. China

H. Sun

School of Science and Ministry of Industry and Information Technology, Key Laboratory of Micro-Nano Optoelectronic Information System, Harbin Institute of Technology (Shenzhen), Shenzhen, Guangdong 518055, P. R. China

J. Qin

Sauvage Laboratory for Smart Materials, School of Materials Science and Engineering, Harbin Institute of Technology (Shenzhen), Shenzhen 518055, China

L. Dang, G.Wang

Shenzhen Key Laboratory for Advanced Materials, School of Materials Science and Engineering, Harbin Institute of Technology (Shenzhen), Shenzhen 518055, P. R. China

X. Xu

National Key Laboratory of Laser Spatial Information, Harbin Institute of Technology (Shenzhen), Shenzhen 518055, P. R. China

Guangdong Provincial Key Laboratory of Aerospace Communication and Networking Technology, Harbin Institute of Technology (Shenzhen), Shenzhen 518055, P. R. China

**S1.** **Transient reflectivity curves of three samples at a low pump fluence.**

Figure S1: Transient reflectivity curves of three samples at a low pump fluence. The blue, green and yellow dots represent the three different samples with different thickness via different systhetic methods. The solid lines represent the corresponding single exponential fitting.

At a low pump fluence, the decay times of  Sample C1, Sample O1, and Sample O2 are 140 ps, 79.65 ps, and 227 ps, respectively.

**S2. Sample uniformity.**

Figure. S2 AFM image of (a) 22.44 nm-thick grown by CVD, (b) 25.1 nm-thick synthesized via OTG solution method and (c) 115 nm-thick synthesized via OTG solution method Bi_2_O_2_Se thin films. The (d), (e) and (f) show the roughness of the samples surface corresponding to the (a), (b) and (c). The roughness data corresponds to the position of the short white line in the AFM image.

In the sample grown by CVD, the surface fluctuation does not exceed 5 Å, while in the samples synthesized by OTG solution method, the surface fluctuation does not exceed 10 Å. AFM measurements show surface fluctuations below 10 Å for all OTG samples, indicating comparable surface morphology. Therefore, surface roughness is unlikely to account for the large lifetime difference between O1 and O2.

Figure. S3 Three-spot measurements for OTG sample with the thickness of 25.1 nm under the fluence of 3.6 mJ/cm^2^.
